# Informed participation? An investigation of the relationship between exposure to different news channels and participation mediated through actual and perceived knowledge

**DOI:** 10.3389/fpsyg.2023.1251379

**Published:** 2024-01-04

**Authors:** Svenja Schäfer, Christian Schemer

**Affiliations:** ^1^Department of Communication, University of Vienna, Vienna, Austria; ^2^Department of Communication, Johannes Gutenberg University Mainz, Mainz, Rhineland-Palatinate, Germany

**Keywords:** news consumption, knowledge, perceived knowledge, participation, illusion of knowledge, social media

## Abstract

Previous studies have found that different media channels have varying potentials for informed participation. Exposure to newspaper and TV news exposure has been shown to increase actual knowledge and participation, while social media is associated with participation based on perceived knowledge, without changes in actual knowledge. In light of these findings, we conducted an online survey (*N* = 1,670) in Germany to investigate the (mediated) relationships between news consumption, knowledge (perception), and participation. The study revealed that exposure to quality newspaper and public service TV news was linked to both actual and perceived knowledge, thereby impacting participation. However, tabloid newspapers and news from private TV channels were not found to be related to participation. In the case of social media, we found a relationship with online participation on social media and offline participation, but this relationship was only mediated through an increase in perceived knowledge. In other words, our findings suggest that social media use creates an illusion of knowledge that is linked to higher levels of participation. These findings highlight the democratic importance of traditional media channels, particularly public service broadcasting media. They also demonstrate how social media can lead to a false feeling of knowing, which can hinder participation processes.

## Introduction

Citizen participation is a key feature of healthy democracies (Delli Carpini and Keeter, 1996; Norris, 2000), as it increases trust in democratic processes (Dahlberg, 2010) and reduces political apathy (Norris, 2000). However, for participation to be effective, citizens must have a clear understanding of their political and social preferences. News media play a vital role in providing the information necessary for informed political decision-making. Therefore, the relationship between news consumption, knowledge, and participation is of a high normative value. It is commonly argued that the link between news consumption and participation is mediated by *objective knowledge* (Jung et al., 2011; Andersen et al., 2016; Lee et al., 2021). For example, a newspaper article discussing tax increases may motivate readers to participate in a demonstration. Conversely, if people are unaware of the tax reform, they are less likely to participate. Numerous studies confirm this mediated relationship between news consumption and participation through knowledge (Jung et al., 2011; Andersen et al., 2016). However, the extent of this relationship depends on the type of news being considered, as different news sources have varying potential for promoting learning (Andersen et al., 2016). In summary, high-quality newspaper articles (both online and offline) and TV news have positive effects on political knowledge (Robinson and Davis, 1990; Dalrymple and Scheufele, 2007) and contribute to informed participation (e.g., Andersen et al., 2016). On the other hand, the use of tabloid news and social media news is less likely to promote participation through increased knowledge (Lee et al., 2021).

Another line of research suggests that news consumption not only alters participation through objective knowledge but also through *knowledge perception*, which refers to what individuals believe they know. Competitive tests of actual and perceived knowledge as mediators for participation reveal that perceived knowledge is even more important than recalled facts (Schäfer, 2020; Lee et al., 2021). Additionally, different types of news have different effects on perceived knowledge. Studies show that easy-to-process information, such as TV news and news on social media, is positively related to perceived knowledge (Lee et al., 2021) even though its potential for promoting learning is limited. Therefore, certain types of news can contribute to informed participation, while others may encourage participation based solely on the feeling of being informed.

The current study aims to investigate how exposure to news from newspapers (both online and offline), TV (both public service broadcasting and private news channels), and social media are related to participation through both actual and perceived knowledge. Previous studies have examined the relationship between news consumption, both actual and perceived, and participation, but these studies were either conducted in experimental settings (Schäfer, 2020) or focused solely on social media as a news source in the US (Lee et al., 2021). As a result, our study contributes to the existing literature by shedding light on the relationship between different news sources and informed or uninformed participation, with a specific focus on public service media, which play an important role as news sources in the European context (Curran et al., 2009). Furthermore, we also differentiate between online participation through social media and offline participation, providing a more nuanced perspective compared to previous studies. In sum, our findings are relevant for two main reasons. Firstly, by considering both actual and perceived knowledge as mediators for participation, we gain a better understanding of citizens’ motivations to engage in political activities, ultimately leading to a better understanding of the motivational and cognitive conditions necessary for a healthy democracy. Secondly, by contrasting actual and perceived knowledge, we can discern which news media are associated with informed participation and which are linked to participation based solely on perceived knowledge, even when actual knowledge may be lacking.

### News consumption and knowledge acquisition

An informed society is crucial for the functioning of a democracy (Delli Carpini and Keeter, 1996; Dutwin, 2003). Without being informed, citizens lack the necessary knowledge to understand their needs and duties as citizens and to make political decisions in line with their interests (Popkin and Dimock, 1999). Political news provides citizens with the necessary information to acquire knowledge, make informed decisions, and participate accordingly. Therefore, the characteristics of available news and news consumption play a vital role in democracies (Woodstock, 2014).

In light of the normative function of being informed, political knowledge is defined as a multidimensional concept that usually distinguishes between *factual knowledge* and *structural knowledge*. Factual knowledge refers to information about textbook facts concerning political structures or knowledge about current affairs (i.e., surveillance knowledge Moeller and de Vreese, 2015). On the other hand, structural knowledge describes a more complex form of knowledge that involves understanding and sense-making of politics by combining the meaning of various facts (Eveland et al., 2004).

When it comes to learning from the news, it is a complex interplay between the supply and demand side (Shehata and Strömbäck, 2021). On the supply side, several content features of news significantly impact the learning process outcome. Firstly, the volume of available information is crucial (Schäfer, 2020). Secondly, the level of complexity also plays a significant role (Tolochko et al., 2019). Lastly, the style of news presentation, whether it is conveyed through textual or visual means, also contributes to the learning process (Lang et al., 2003). A high volume of relevant information presented in a text-based manner, with a sufficient level of complexity, promotes activation and an elaborated style of news processing which fosters learning processes (Robinson and Davis, 1990). On the demand side, individuals initiate learning by exposing themselves to news (Castro et al., 2021), and devoting cognitive capacity to the elaboration and processing of information (Eveland, 2001). However, the supply and demand side interact, as content features can influence information processing styles and some news environments can be more distracting than others (Lang et al., 2003). This explains why different types of news have different potentials for learning.

Based on this reasoning and considering the relevant content features, it is prudent to analyze quality newspapers and tabloid newspapers separately due to their notable differences. Quality newspapers, both online and offline, have a high potential for learning. They offer a substantial volume of information presented in a complex yet understandable manner through textual information. These attributes prove advantageous for achieving positive learning outcomes as they require both activation and an elaborated style of information processing (Robinson and Davis, 1990). Indeed, several studies confirm a positive relation between exposure to news articles offline (Fraile and Iyengar, 2014) and online (Dalrymple and Scheufele, 2007; Dimitrova et al., 2011; Andersen et al., 2016) with political knowledge. On the other hand, tabloid press news articles have less potential for learning about politics. This can be explained by the typical content features of tabloid media: they have a lower volume of political news, present information in a less complex manner due to a stronger focus on entertaining features, and provide higher shares of visual content, which lowers opportunities for learning (de Vreese and Boomgaarden, 2006). Findings confirm that engaging with news provided by tabloid media does not increase political knowledge (Horstmann, 1991; Newton, 1999; Fraile and Iyengar, 2014).

When examining the potential of TV news for learning, it is essential to distinguish between news delivered by public service broadcasting and private TV networks, as they exhibit variances in terms of the relevant content features. Their common feature is that they provide visual content which comes with a lower density of information compared to text-based information (Robinson and Davis, 1990) and fosters more superficial and passive cognitive engagement, hindering learning processes (Becker and Whitney, 1980; Grabe et al., 2009). In general, the potential for learning is lower compared to news provided by newspapers. However, content analyses show that news from public service broadcasters air high-quality news programs, in terms of both information density and news formats (Curran et al., 2009; Aalberg et al., 2012). On the other hand, news from private TV networks has a lower potential for learning due to its lower information density and reduced complexity. This explains the finding that the relation between TV news consumption and knowledge is stronger in countries with a public service broadcasting system (Curran et al., 2009; Iyengar et al., 2009; Shehata et al., 2015; Park and Gil de Zúñiga, 2021). For example, Strömbäck (2016) compared the effects of watching public service news and news on commercial TV and found that watching news from public service broadcasters has a positive effect on knowledge, while exposure to news on commercial TV programs is even negatively related to knowledge.

Finally, social media has become a unique source of political information that has gained popularity among citizens, especially younger people (Newman et al., 2022). Unlike newspapers and TV news broadcasts, social media ranks poorly in terms of content features relevant to learning, as it primarily presents news in a bite-sized, snackable format (Schäfer, 2020). This means that the information density is minimal and presented in a simplified format to facilitate quick consumption amidst a backdrop of friend updates, entertainment pages, and advertisements (Kümpel, 2021). Even if the news is from high-quality sources, the potential of social media for learning about politics is limited due to the short length of textual posts and videos, and the distracting information environment. Occasionally, users might click on posts they come across in their news feed, leading to a news consumption episode with more substantial information. However, research indicates that only a small fraction (7%) of the news content discovered on social media is actually clicked, reaffirming that brief news posts continue to serve as the primary source of information (Bakshy et al., 2015). Results confirm that engaging with news on social media has no statistical association with knowledge (Dimitrova et al., 2011; Ran et al., 2016; Au et al., 2017; Feezell and Ortiz, 2019) or even a negative relation with political learning (Cacciatore et al., 2018; Lee et al., 2021; Shehata and Strömbäck, 2021). However, there are also findings that show that people remember information they found on social media (Bode, 2016) and can learn about politics, but only if they click on the news posts (Anspach et al., 2019; Nanz and Matthes, 2020; Park and Gil de Zúñiga, 2021).

In sum, it can be concluded that newspaper articles, TV news, and news on social media have different content characteristics. They also foster varying degrees of cognitive engagement, which ultimately lead to different outcomes in terms of learning and political knowledge. Therefore, we assume

*H1*: Exposure to news articles (online and offline) in quality newspapers is positively related to political knowledge.

*RQ1*: What is the relationship between exposure to news articles (online and offline) in tabloid media and political knowledge?

*H2*: Exposure to public service TV news is positively related to political knowledge.

*RQ2*: What is the relationship between exposure to commercial TV news and political knowledge?

*RQ3*: What is the relationship between exposure to news on social media and political knowledge?

### News consumption and perceived knowledge

Considering a metacognitive perspective on knowledge, it is important to differentiate between objective knowledge and perceived knowledge (Nelson and Narens, 1990). Objective knowledge refers to information stored in long-term memory that people can recall or explain (Raaijimakers and Shiffrin, 1980), which we previously described as factual or structural knowledge. On the other hand, perceived knowledge refers to the awareness people have about their knowledge (Nelson and Narens, 1990). This metacognitive perception of knowledge is not tied to specific information that people remember, but rather it is an intuition that spontaneously comes to people’s minds when they think about what they know (Nelson and Narens, 1990).

While activation (Ashcraft and Radvansky, 2010) and elaboration (Craik and Lockhart, 1972) determine how well people can remember information and thus influence objective knowledge, the sources for knowledge perception are different. Familiarity with a certain topic (Metcalfe et al., 1993) and the ease of processing information about that topic are important for perceived knowledge (Scharrer et al., 2017; Ryffel and Wirth, 2020). Put differently, if people frequently encounter a topic and can easily understand the information they receive, they tend to think they know a lot about that topic.

When applying the content features that are relevant to objective knowledge to the process of knowledge perception, it becomes apparent that they also influence what individuals perceive to know. However, this perception may not always align with their actual learning outcomes. Firstly, although a high information density is beneficial for learning, it may not necessarily positively influence individuals’ perception of what they know. This is because it provides people with a more accurate understanding of the complexity of a news topic. Secondly, when it comes to the complexity of news, people tend to think they know more about a news topic if it is presented to them in an easy-to-process way. Thirdly, while text is more beneficial for learning compared to visual content, it is not necessarily related to higher levels of knowledge perception.

Reading newspaper articles, for example, requires higher levels of cognitive effort due to the high density of text-based information. This higher effort can result in lower ease-of-processing perceptions and consequently lower or only moderate knowledge perceptions. An experiment found that reading a newspaper article did not cause any change in knowledge perception compared to a control group that did not receive any information (Schäfer, 2020). People reading newspaper articles may also be humbler than non-readers, as they are aware that they only read part of the articles and there is more they could potentially learn from. Consequently, perceived knowledge may be lower than their current knowledge. However, the relationship between subjective knowledge and reading newspaper articles, both quality press and tabloid media, has hardly been investigated in previous studies.

TV news, on the other hand, offers visual content that has a lower density of information and is presented in a less complex way compared to newspaper articles. While this may not be as favorable for learning, it can have a positive effect on knowledge perception. Studies have shown that watching news on TV is positively related to perceived knowledge (Park, 2001; Mattheiß et al., 2013; Ryffel and Wirth, 2020). This can be explained by the entertaining character of the easy-to-process visual content provided on TV which increases perceived fluency and consequently perceived knowledge (Ryffel and Wirth, 2020).

Finally, while characteristics of news on social media have low potential for learning, they can have a positive influence on knowledge perception. News posts on social media are usually short, contain visual content (such as a picture or video), and have catchy headlines to persuade users to click on the post. These features make them easy to process, which can increase the impression that people have a good understanding of the presented topic (Schäfer, 2020). However, social media users often miss out on nuances of a news topic because they predominantly remain on social network sites and do not follow links to full-length news stories. This phenomenon contributes to the overestimation of their knowledge about a given news topic. Moreover, within social media environments, topics are frequently repeated, either because the same topic is picked up by several media outlets or due to algorithms that repeatedly confront users with similar topics (Thorson, 2020). This increases familiarity with a news topic which, in turn, increases perceived knowledge (Metcalfe et al., 1993). Several studies have shown that even though the effects of social media news on actual knowledge are limited, it increases the feeling of being informed (Ran et al., 2016; Anspach et al., 2019; Schäfer, 2020; Lee et al., 2021).

Therefore, the following research questions and hypotheses for news media exposure and knowledge perception can be derived.

*RQ4*: How is exposure to (a) news articles in quality press and (b) news articles in the tabloid press related to perceived knowledge?

*H3*: Exposure to (a) public service TV news and (b) commercial TV news is positively related to perceived knowledge.

*H4*: Exposure to news on social media is positively related to perceived knowledge.

### Actual and perceived knowledge as mediators for participation

Civic participation can be broadly defined as communicative acts of opinion and expressing one’s will (Barber, 1984). This can include participating in protests, supporting political parties, or endorsing a political candidate (Theocharis and van Deth, 2018). The rise of Web 2.0 applications has greatly expanded the possibilities for participation (Dahlberg, 2010). Particularly through social media platforms. These platforms provide an infrastructure where users can share their political views, support political actors or parties, and directly communicate with politicians (Rowe, 2015). It is important to note that offline and online participation, specifically on social media, not only differ in their contexts (virtual vs. real-life) but also in terms of the costs involved (Friess and Eilders, 2015).

When examining the relationship between news consumption and participation, a plethora of studies have confirmed a positive correlation (e.g., Ksiazek et al., 2010; Boulianne, 2015; Edgerly et al., 2018; Geers and Vliegenhart, 2021). However, it is also argued that news consumption indirectly affects participation. Objective knowledge is often seen as a mediator in this relationship. Several studies have found that news consumption is related to participation through the knowledge people have about a particular topic (Delli Carpini and Keeter, 1996; Kenski and Stroud, 2006; Jung et al., 2011; Andersen et al., 2016). This aligns with the normative perspective of a citizenry making informed political decisions (Delli Carpini and Keeter, 1996).

However, politically motivated behavior can also be influenced by what individuals believe they know about politics. For instance, many people choose not to participate in elections because they think to lack sufficient knowledge to make an informed voting decision (Kaid et al., 2007; European Commission, 2009). Other findings suggest that individuals with higher levels of perceived knowledge are more likely to engage in political discussions (Schäfer, 2020), and participate in both online (Yamamoto et al., 2018) and offline activities (Lee and Matsuo, 2018; Lee et al., 2021). In fact, a comparison of the (mediated) effects of actual and perceived knowledge on participation revealed that perception of knowledge is more relevant than performance in knowledge tests (Lee and Matsuo, 2018; Schäfer, 2020; Lee et al., 2021).

In sum, news media have different relationships with actual and perceived knowledge, leading to different indirect relationships with participation. Given the aim of our study to explore these indirect relationships with participation, we have formulated the following research questions:

*RQ5*: How are different types of news media related to (a) offline participation and (b) online participation on social media through objective knowledge?

*RQ6*: How are different types of news media related to (a) offline participation and (b) online participation on social media through knowledge perception?

## Methods

### Data

To address our research questions and test our hypotheses, we employed data obtained from an online survey conducted in Germany as part of a larger project. This survey was designed to create a sample representative of the German population and was carried out by the professional market research company, Dynata, in December 2019. Notably, this data collection occurred prior to the onset of the COVID-19 pandemic in Germany in March 2020.

In order to ensure the representativeness of our sample, we applied quotas for age, gender, and education during the participant recruitment process. The responsibility for data collection and subsequent data cleaning was entrusted to the market research company. As part of the data cleaning procedure, only those participants who completed the survey within a reasonable time frame and successfully passed an attention check, which involved selecting a specific item from an item scale, were included in the final sample. The final sample consists of *N* = 1,670 participants, of which 53% were female and 22% had a college degree. The mean age is 44 years (*SD* = 12.68). The sample does not represent the German voting age population since people with higher educational degrees are overrepresented (the questions used for this study, the data set, and syntax can be found at: https://osf.io/kze5h/?view_only=1b0495b5fcef4d55be4e5f2b10875325).

The present study is without any interventions or disturbing elements. Therefore, it was not reviewed by an ethics board. This is also not required by the federal law in which the study was conducted.

### Measures

#### News consumption

To measure news consumption, we examined the most frequently used news outlets in Germany (Hölig et al., 2019; Zubayr and Gerhard, 2019). Participants were asked to indicate how often they followed the news on these outlets in an average week using a verbalized scale ranging from 0 = never to 6 = several times a day. For *quality newspapers,* we focused on the Süddeutsche Zeitung and Frankfurter Allgemeine Zeitung and calculated a sum index for these newspapers (*M* = 1.44, *SD* = 2.32). For *tabloid newspapers*, participants reported their usage of BILD and local boulevard newspapers (e.g., BZ, Kölner Express, etc.). Again, we created a sum index based on these two variables (*M* = 1.72, *SD* = 2.47). To measure consumption of *public service TV news*, we asked participants how often they followed the news on ZDF and ARD, combining their responses to form a sum index (*M* = 5.19, *SD* = 3.33). For *commercial TV programs*, we considered news on RTL and SAT1, and combined the answers to these items (*M* = 3.36, *SD* = 2.95). Finally, we asked participants about their frequency of news consumption on *social media platforms* (e.g., Facebook, Twitter, Instagram) in an average week (*M* = 2.49, *SD* = 2.31).

#### Objective knowledge

To assess levels of objective knowledge we developed a knowledge test consisting of seven questions covering a wide range of political topics, including political actors, policies, and current affairs, at both national and international levels. The questions were multiple-choice questions, with participants having four response options to choose from. Additionally, participants were provided with a “do not know” option for each question. For analysis purposes, we recoded all answers for the knowledge questions as either 0 = wrong answer/do not know, or 1 = correct answer, and summed them up to create an index (*M* = 3.72, *SD* = 1.49). A complete list of the knowledge questions and the answering options can be found in the Appendix.^1^

#### Perceived knowledge

To assess perceived knowledge, we used a single item stating “Compared to most people, I know a lot about politics.” Participants were asked to indicate their level of agreement on a scale ranging from 1 = completely disagree, to 7 = completely agree (*M* = 4.25, *SD* = 1.68).

### Participation

For the measurement of participation, we utilized the items provided by Theocharis and van Deth (2018) to distinguish between offline and online political participation on social media. *Offline participation* encompassed 10 activities such as campaigning (e.g., donating money to a political party), civic participation (e.g., donating money to a non-profit organization), and protests (e.g., participating in a march). Participants indicated the frequency with which they engaged in these activities over the past 12 months using a scale ranging from 1 = never, 2 = rarely, 3 = from time to time, to 4 = often. The scores for all 10 items were summed to create an index (*M* = 15.77, *SD* = 5.60). For *online participation*, we inquired about the frequency of participants’ engagement with political posts on social media, including liking, sharing, or commenting. Moreover, we asked if they followed any politicians and whether they had changed their profile picture to make a political statement. The same 4-point scale was employed for these online participation items (*M* = 7.67, *SD* = 3.96).

### Data analysis

**Figure 1:**
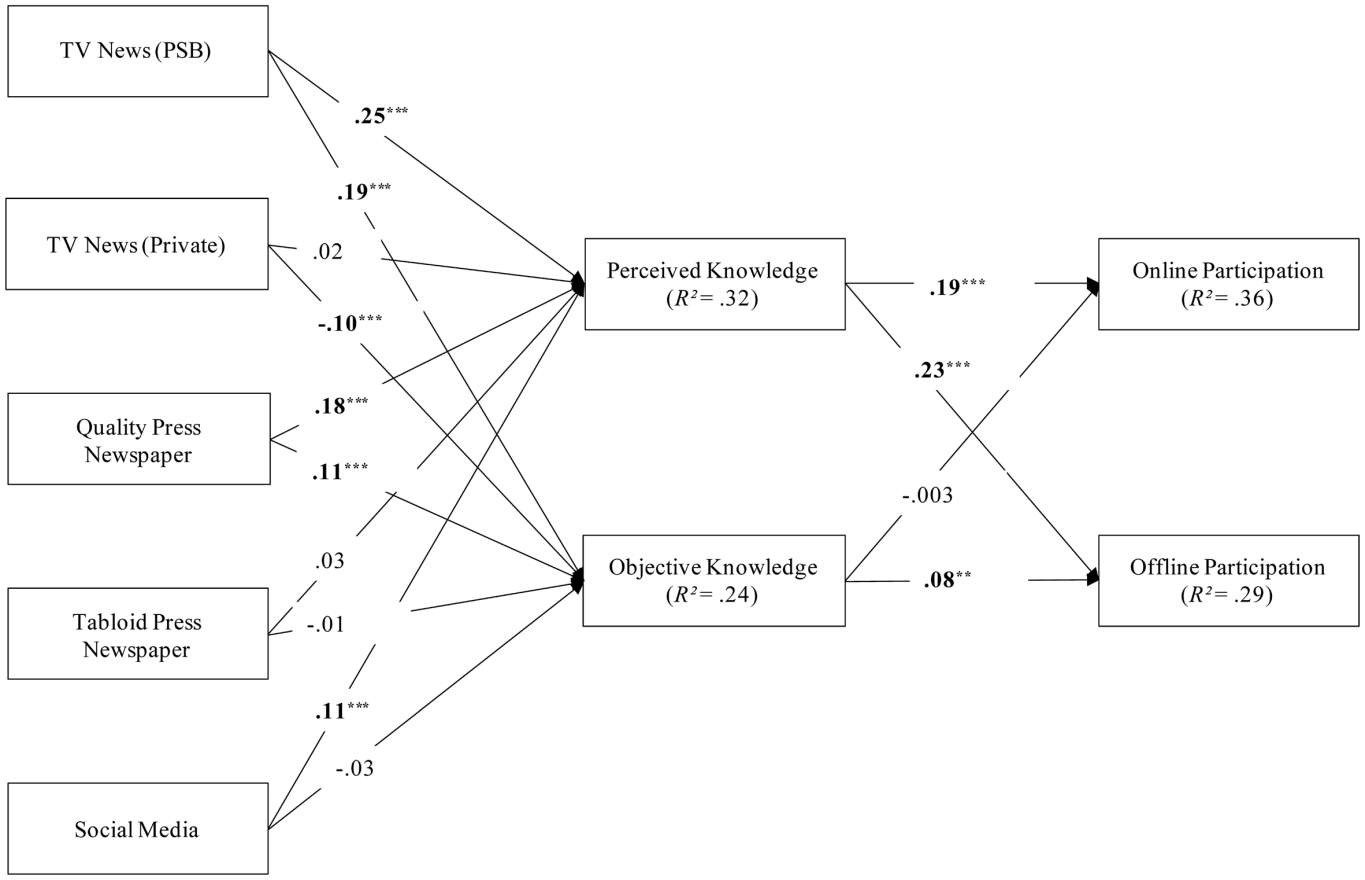
Summary of the findings. *n* = 1,423. Coefficients are standardized. All paths are controlled for age, gender, and education. ^†^*p* < 0.10, ^*^p < 0.05, ^**^p < 0.01, ^***^p < 0.001. The relationship between exposure to different news channels and participation mediated through actual and perceived knowledge.

To analyze the relationship between news consumption, actual and perceived knowledge, and online and offline participation, we used the R package lavaan (Rosseel, 2012) to calculate a path model. The model estimated direct paths between news consumption and both actual and perceived knowledge, as well as indirect paths between news consumption and participation through the two types of knowledge. We included every possible path in the model, resulting in the absence of degrees of freedom and the unavailability of model fit indices. Figure 1 provides a summary of the findings. All indirect paths are described in the text and were tested using a 95% bias-corrected confidence interval (CI) with 5,000 bootstrap subsamples.

## Results

### News consumption and objective knowledge

In the first hypothesis and research question, we examined the relationship between exposure to quality newspapers, tabloid newspapers, and objective knowledge. The results of the path model indicate that the use of quality press, both online and offline, was positively correlated with knowledge (*ß* = 0.11, *p* < 0.001). However, there was no correlation between tabloid press news use and knowledge (*ß* = −0.01, *p* = 0.636). Thus, H1 is not rejected. Moving on to H2, we found a positive association between exposure to news on public service broadcasting and knowledge (*ß* = 0.19, *p* = 0.001), indicating that H2 is not rejected. RQ2 investigates the relationship between commercial TV news consumption and political knowledge. We found a negative relationship in this case (*ß* = −0.10, *p* < 0.001). Finally, in regards to RQ3, which explores the relationship between social media news consumption and knowledge, we found no significant association (*ß* = −0.03, *p* = 0.272).

### News consumption and perceived knowledge

The fourth research question examined the relationship between exposure to news in quality press and tabloid press and perceived knowledge. We found a positive relationship for quality newspapers (*ß* = 0.18, *p* < 0.001), while tabloid newspaper use was not associated with perceived knowledge (*ß* = 0.03, *p* = 0.35). We assumed a positive association between perceived knowledge and public service news, as well as commercial TV news. Partially confirming this assumption, we found a positive relationship for public service news use (*ß* = 0.25, *p* < 0.001), while watching commercial news was not related to knowledge perception (*ß* = 0.02, *p* = 0.397). Additionally, the exposure to news on social media was also positively related to perceived knowledge (*ß* = 0.11, *p* < 0.001), indicating that H4 is not rejected.

### Mediated effects

The results for the indirect paths show that exposure to *quality newspapers* was related to offline participation through both objective (*ß* = 0.01, *p* < 0.001) and perceived knowledge (*ß* = 0.04, *p* < 0.001). Quality newspapers were also directly positively related to offline participation (*ß* = 0.29, *p* < 0.001). There was no mediated relationship between quality newspaper use and online participation on social media through actual knowledge (*ß* = -0.000, *p* = 0.912). However, the indirect path through perceived knowledge achieved statistical significance (*ß* = 0.03, *p* < 0.001). Additionally, quality newspapers were positively related to online participation on social media (*ß* = 0.24, *p* < 0.001). In sum, quality newspaper use was related to both online and offline participation, both directly, and through increases in actual and perceived knowledge.

For *tabloid media*, there was no significant indirect relation with offline participation, neither via actual (*ß* = −0.001, *p* = 0.660) nor perceived knowledge (*ß* = 0.006, *p* = 0.350). However, there was a positive direct relationship (*ß* = 0.18, *p* < 0.001). The same pattern can be found for online participation on social media, which was also not indirectly related (perceived knowledge: *ß* = 0.005, *p* = 0.354; actual knowledge *ß* = 0.00, *p* = 0.961) to tabloid news exposure. However, there was a positive direct relationship between tabloid newspaper exposure and online participation (*ß* = 0.41, *p* < 0.001). This means that tabloid media is only directly related to offline and online participation on social media, but not through changes in knowledge or knowledge perception.

For *public service TV news*, we found a significant indirect relation with offline participation through actual knowledge (*ß* = 0.016, *p* < 0.01) and perceived knowledge (*ß* = 0.06, *p* < 0.001). The direct relationship between public service TV news and offline participation did not reach significance (*ß* = 0.05, *p* = 0.142). For online participation on social media, there was no significant indirect relation via actual knowledge (*ß* = −0.001, *p* = 0.910), but through perceived knowledge (*ß* = 0.05, *p* < 0.001). The direct relationship was non-significant (*ß* = −0.03, *p* = 0.289).

*Commercial TV news* was negatively related to offline participation through actual knowledge (*ß* = −0.009, *p* < 0.05), while there was no indirect relation via perceived knowledge (*ß* = 0.005, *p* = 0.41). There was also no direct relationship between commercial TV news and offline participation (*ß* = −0.06, *p* = 0.128). For online participation, there was no indirect relation of commercial news through actual knowledge (*ß* = 0.000, *p* = 0.961), nor through perceived knowledge (*ß* = 0.005, *p* = 0.926). Additionally, the direct relationship between commercial TV news and online participation on social media was not significant (*ß* = 0.04, *p* = 0.161).

Finally, we found that *social media news* consumption was not indirectly related to offline participation through actual knowledge (*ß* = −0.002, *p* = 0.336), but through perceived knowledge (*ß* = 0.03, *p* < 0.001). There was also a direct positive relationship between social media news and offline participation (*ß* = 0.26, *p* < 0.001). The same pattern can be found for online participation on social media which was not indirectly related to social media news consumption through actual knowledge (*ß* = 0.000, *p* = 0.934), but through perceived knowledge (*ß* = 0.02, *p* < 0.001). Here, the direct relationship also reached statistical significance (*ß* = 0.50, *p* < 0.001), indicating that social media is related to online and offline participation through what people think they know and through a direct relationship.

## Discussion

Previous research suggests that different media channels have varying potentials for learning and political engagement based on knowledge. Additionally, media use is also connected to participation through its impact on perceived knowledge. Thus, while some media channels likely contribute to an informed participation based on actual knowledge, other channels might contribute to participation through a feeling of being informed. Therefore, this study aimed to test the direct relationships between different news channels, and actual and perceived knowledge as well as indirect relationships between media use and participation.

The findings for actual knowledge align with previous research on news consumption and knowledge. Exposure to quality newspapers is positively related to objective knowledge, while exposure to tabloid newspapers has no such relationship. The association between TV news use and objective knowledge depends on the specific program. The present findings show that exposure to news provided by public service broadcasters is positively related to knowledge while using commercial news programs is even negatively related to knowledge. Furthermore, social media consumption for news is not related to actual knowledge, which is consistent with previous studies (Ran et al., 2016). In sum, the results confirm that the use of news channels that provide in-depth information on a broad range of political topics is also positively associated with objective knowledge. On the other hand, news that is too entertaining, superficial, focused on soft news, or presented in information environments without a focus on news is not related to the knowledge of viewers, listeners, or readers.

Considering perceived knowledge, it appears that the audience of TV news and newspapers can accurately estimate their level of political knowledge. For example, heavy users of quality press have higher levels of knowledge and also feel more knowledgeable. However, this is different for social media news use. Frequent use of social media for news does not result in higher levels of actual knowledge, but it is associated with feeling more knowledgeable. This finding aligns with previous research on the illusion of knowledge through social media news consumption (Ran et al., 2016; Schäfer, 2020; Lee et al., 2021). News posts on Instagram or videos on TikTok provide easily digestible information with an entertaining character, which can positively impact knowledge perception. However, social media posts have a low information density and appear in distracting environments, limiting their potential for actual learning.

Furthermore, the path model shows that perceived knowledge has a stronger relationship with both online, and offline participation compared to actual knowledge. This may be because actual knowledge provides the necessary information to understand the complexity of political topics, leading to a more modest perception of knowledge (Fernbach et al., 2013). In terms of media channels, only news from public service broadcasting and quality press are indirectly related to offline participation through actual learning. This suggests that these news channels contribute to informed participation, highlighting their value for democratic processes. On the other hand, commercial TV news and tabloid press are not indirectly related to participation through perceived knowledge, or can even have a negative relationship with participation due to lower actual knowledge. Therefore, they do not play a role in informed or uninformed participation. Social media, however, is related to both offline and online participation through an increase in perceived knowledge. This means that even though social media use for news is not related to objective knowledge, it is linked to the feeling of being informed, which indirectly impacts political activities online and offline. Assuming that social media encourages participation, this outcome is desirable because citizens need to engage in public will formation processes (Dahlberg, 2010). However, social media news consumption is not related to political learning, indicating that participation fostered by social media may be based on an insufficient understanding of current affairs, potentially hindering an informed public discourse.

Naturally, our study comes with limitations. In interpreting our findings, it is essential to acknowledge that our results stem from the analysis of cross-sectional survey data. Due to the inherent characteristics of such data, we are unable to empirically test the theoretical model we have constructed based on prior research. Consequently, we cannot definitively eliminate the possibility that the arrangement of constructs in our model, causality, or even potential confounding variables that are pivotal for the pathways we have delineated may differ from our current modeling (Rohrer et al., 2022). While we have established empirical relationships between certain constructs in our model, it is important to note that even though our model aligns with previous findings, establishing a causal relationship would necessitate the use of either experimental or longitudinal data.

Another limitation concerns the measurement of actual and perceived knowledge. Actual knowledge was measured using seven multiple-choice questions covering a broad range of political facts. However, it is unknown if this information was currently part of the news coverage during the data collection period or if it was equally presented in the different channels investigated. As a result, the different news channels may have increased different types of political knowledge that were not covered by the questions asked. Perceived knowledge was assessed using a single-item measure, which has limitations in terms of reliability. This should be improved in future studies.

In conclusion, quality press and public service broadcasters may play a crucial role in promoting an informed public discourse. They are associated with higher levels of knowledge, which in turn is related to participation, highlighting their normative value. While the study cannot establish a causal relationship between news media exposure, knowledge, and participation with the data used, the content features of traditional media and empirical findings support the conclusion that traditional media plays a vital role in knowledge acquisition and should not be replaced by social media formats. The use of platforms and social network sites are related to online and offline participation, but only through perceived knowledge, not actual knowledge. It is important to recognize the potential of social media as a news channel, as it provides opportunities to learn about current topics and entertainingly engage with politics. However, to develop an understanding of current affairs and to gain a nuanced perspective on political issues, social media should serve as gateways to news or be an addition, rather than a substitute, for a news diet that includes sources from traditional outlets.

## Data availability statement

The datasets presented in this study can be found in online repositories. The names of the repository/repositories and accession number(s) can be found in the article/supplementary material.

## Ethics statement

Ethical approval was not required for the studies involving humans because this was not required by the federal law as the research topic was not sensitive. The studies were conducted in accordance with the local legislation and institutional requirements. The participants provided their written informed consent to participate in this study.

## Author contributions

SS was in charge of the theory, the setup of the model, and the writing of the paper. CS was in charge of the data collection and the statistical modeling. SS and CS worked closely together for this paper and double-checked, commented and improved all parts. All authors contributed to the article and approved the submitted version.

## Footnotes

^1^ https://osf.io/kze5h/?view_only=1b0495b5fcef4d55be4e5f2b10875325
